# A Digital Health Platform for Integrated and Proactive Patient-Centered Multimorbidity Self-management and Care (ProACT): Protocol for an Action Research Proof-of-Concept Trial

**DOI:** 10.2196/22125

**Published:** 2021-12-15

**Authors:** John Dinsmore, Caoimhe Hannigan, Suzanne Smith, Emma Murphy, Janneke M L Kuiper, Emma O'Byrne, Mary Galvin, An Jacobs, Myriam Sillevis Smitt, Cora van Leeuwen, Patricia McAleer, Lorraine Tompkins, Anne-Marie Brady, Mary McCarron, Julie Doyle

**Affiliations:** 1 Trinity Centre for Practice and Healthcare Innovation School of Nursing and Midwifery Trinity College Dublin Dublin Ireland; 2 School of Computer Science Technical University Dublin Ireland Ireland; 3 NetwellCASALA Dundalk Institute of Technology Dundalk Ireland; 4 Imec-VUB-SMIT Brussels Belgium; 5 Home Instead Senior Care Dublin Ireland; 6 School of Applied Psychology University College Cork Cork Ireland; 7 Department of Design Innovation Faculty of Social Sciences Maynooth University Maynooth Ireland; 8 Trinity Centre for Ageing and Intellectual Disability School of Nursing and Midwifery Trinity College Dublin Dublin Ireland

**Keywords:** multimorbidity, digital health, chronic disease, self-management, older adults, integrated care, behaviour change, mobile phone, smart phone, smart device

## Abstract

**Background:**

Multimorbidity is defined as the presence of two or more chronic diseases and associated comorbidities. There is a need to improve best practices around the provision of well-coordinated, person-centered care for persons with multimorbidities. Present health systems across the European Union (EU) focus on supporting a single-disease framework of care; the primary challenge is to create a patient-centric, integrated care ecosystem to understand and manage multimorbidity. ProACT is a large-scale project funded by the European Commission under the Horizon 2020 programme, that involved the design, development, and evaluation of a digital health platform to improve and advance home-based integrated care, and supported self-management, for older adults (aged ≥65 years) living with multimorbidity.

**Objective:**

This paper describes the trial implementation protocol of a proof-of-concept digital health platform (ProACT) in 2 EU member states (Ireland and Belgium) to support older persons with multimorbidities self-managing at home, supported by their care network (CN).

**Methods:**

Research was conducted across 2 EU member states, Ireland and Belgium. A 12-month action research trial design, divided into 3 evaluation cycles and lasting 3 months each, with a reflective redesign and development phase of 1 month after cycles 1 and 2 was conducted. Participants were 120 (60/120, 50% in Ireland and 60/120, 50% in Belgium) older persons with multimorbidities diagnosed with two or more of the following chronic conditions: diabetes, chronic obstructive pulmonary disease, chronic heart failure, and cardiovascular diseases. With permission from persons with multimorbidities, members of their CN were invited to participate in the study. Persons with multimorbidities were provided with ProACT technologies (tablet, devices, or sensors) to support them in self-managing their conditions. CN members also received access to an app to remotely support their persons with multimorbidity. Qualitative and quantitative feedback and evaluation data from persons with multimorbidity and CN participants were collected across four time points: baseline (T1), at the end of each 3-month action research cycle (T2 and T3), and in a final posttrial interview (T4). Thematic analysis was used to analyze the qualitative interview data. Quantitative data were analyzed via platform use statistics (to assess engagement) and standardized questionnaires (using descriptive and inferential statistics). This study is approved by the ethics committees of Ireland and Belgium.

**Results:**

The trial implementation phase for this 44-month (2016-2019) funded study was April 2018 to June 2019. The trial outcomes are at various stages of publication since 2021.

**Conclusions:**

ProACT aims to co-design and develop a digital intervention with persons with multimorbidities and their CN, incorporating clinical guidelines with the state of the art in human-computer interaction, behavioral science, health psychology, and data analytic methods to deliver a digital health platform to advance self-management of multimorbidity at home, as part of a proactive, integrated model of supported person-centered care.

**International Registered Report Identifier (IRRID):**

RR1-10.2196/22125

## Introduction

### Background

Within the European Union (EU), an estimated 50 million people live with multimorbidity, defined as two or more chronic health conditions [1]. For individuals living with multimorbidity, the self-management of multiple conditions can impose a significant burden [2], with activities that include managing multiple symptoms, medications, information on their conditions, and clinical appointments. In addition, health care services for individuals with multimorbidity are often repetitive (multiple appointments), inconvenient, inefficient (individuals may see different clinicians who give conflicting advice), burdensome, and potentially unsafe due to poorly integrated and coordinated care [3,4]. The outcome for individuals is reduced quality of life, as time and energy are spent managing multiple conditions, limiting their opportunity for social or personal activities [5].

The risk of multimorbidity increases with advancing age, with prevalence rates estimated at 65% in people aged ≥65 years, 85% in people aged ≥85 years, and rising [6]. The rapid aging of the global population brings significant concerns over the sustainability of health services, due to associated increases in health care expenditure, and disparities in the number of practicing health professionals. It is therefore important that efforts are made to explore sustainable digital approaches to support home-based self-management of chronic diseases and multimorbidity. Self-management (or self-care) can be described as an individual’s ability to manage symptoms, treatment, emotions, and lifestyle changes as part of living with a chronic condition [7]. Improving best practices around the provision of person-centered care for a person with multimorbidity requires empowering the Person with multimorbidity to self-manage, actively supported by their care network (CN), which primarily involves informal carers (ICs), formal carers (FCs), and health care professionals (HCPs). The CN of a person with multimorbidity plays an important role in diminishing the impact of disease management, which may subsequently improve health outcomes and quality of life [8].

Digital health technologies have the potential to improve and advance home-based self-management for older persons with multimorbidity, yet most digital solutions focus on single-disease management (eg, diabetes) [9,10]. Therefore, digital solutions that address complex disease management and multimorbidity, taking into account the role, views, and needs of the person with multimorbidity and their CN, are also required.

To date, there has been limited research examining the potential of digital health support for multimorbidity management. This includes understanding the challenges faced by people managing multimorbidity, as well as design requirements for digital technologies to address these challenges [11-15]. Although such research is necessary, to the best of our knowledge, research on digital platforms and systems to support multimorbidity has not progressed beyond examining requirements and suggesting design recommendations.

Within the EU, the ICARE4EU program provides the most robust examination of digital or eHealth use to address multimorbidity management within the context of integrated care [16]. Managers of 101 integrated care programs in Europe were surveyed to understand if they had used eHealth (or digital) solutions and, if so, what were the benefits of and barriers for the solutions in relation to multimorbidity care. Of these programs, 85 adopted eHealth solutions, and 42 of these were targeted specifically at older adults. The types of eHealth technologies implemented within these programs included remote consultation and monitoring, self-management tools (including electronic reminders and web-based decision support), health care management technology such as patient databases and e-referral systems, and electronic health records. However, neither detailed descriptions of these technologies nor their evaluations were presented. Furthermore, the authors noted limitations in that HCPs, patients, and their caregivers were not consulted in terms of the availability of eHealth supports within these programs.

With such limited research in the areas of digital health, integrated care, and multimorbidity management, there is a need for large-scale, longitudinal programs or projects to better understand both the complexities of multimorbidity and how digital technologies can be designed, developed, and implemented to support the person with multimorbidity and their CN. The ProACT project, funded by the European Commission Horizon 2020 programme, brings together a multidisciplinary consortium of 13 European partners for the purpose of developing and evaluating a digital integrated care system to empower home-based, patient-centric care and proactive self-management of chronic conditions for Europe’s 50 million persons with multimorbidity.

This paper reports the protocol for the ProACT Horizon H2020 project main proof-of-concept (PoC) trial conducted in Ireland (by the Trinity Centre for Practice and Healthcare Innovation, Trinity College Dublin, NetwellCASALA at Dundalk Institute of Technology and Home Instead Senior Care) and Belgium (by imec at the Studies in Media, Innovation and Technology in the Vrije Universiteit Brussel) between April 2018 and June 2019. Before the PoC trial, the ProACT platform was designed and developed between 2016 and 2018 through an iterative user-centered process involving input from 166 key stakeholders (older people with multiple chronic conditions, carers, and HCPs) across Ireland, Belgium, and Italy [17-21].

### Study Aim and Objectives

The study evaluated, at a PoC level, a digital health platform (called *ProACT*) for older persons with multimorbidity to self-manage their conditions with support from their CN. ProACT was implemented at 2 EU trial sites (Belgium and Ireland). The specific aims of the trial were (1) to explore the potential benefits of the ProACT platform for persons with multimorbidity and (2) to obtain feedback from all relevant participants on their experiences using the ProACT platform and on the potential of the platform to improve integration of care and support for multimorbidity disease management.

Specific objectives for all participants were to evaluate the usability, accessibility, and acceptability of the ProACT platform, user adoption and satisfaction with the technology and services, and experiences of participants using ProACT. Additional objectives for persons with multimorbidity were to evaluate the potential impact of the ProACT platform on a range of health, well-being, psychological, and psychosocial outcomes and evaluate the efficacy of ProACT as a behavior change intervention that aims to improve self-management skills for the person with multimorbidity. Additional objectives for IC and FC participants were to evaluate the potential impact of the ProACT platform on their psychological and psychosocial outcomes.

### ProACT: Intervention Description

**Figure 1:**
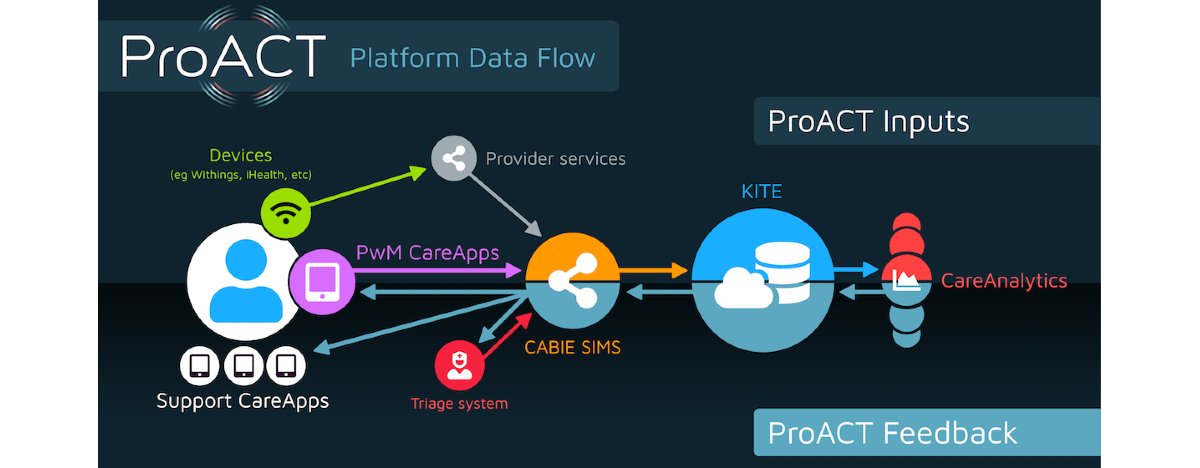
ProACT platform overview and data flow.

**Figure 2:**
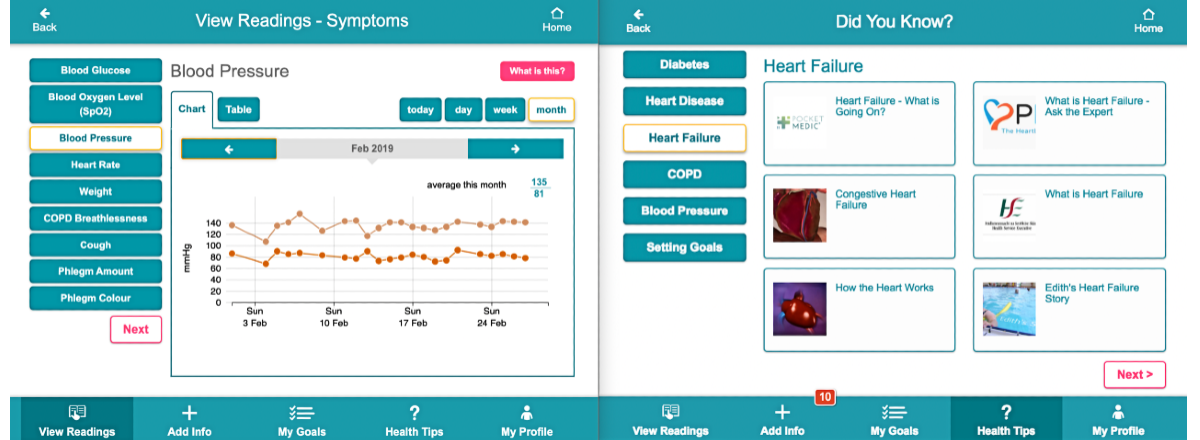
ProACT CareApp View Readings and Did You Know (education) interfaces.

ProACT is a citizen-driven, self-management orientated, digital integrated care platform capable of supporting multiple disease management and well-being parameters (eg, mobility and sleep) on a single user app. The overall platform (Figure 1) consists of the following:

- A kit of home-based health care support tools and
  off-the-shelf
  measurement and sensing devices (eg, blood pressure cuff, weight scales, smart watch, and home-based sensors).
- A suite of end-user apps and support tools (CareApps; Figure 2). Apps are available for persons with multimorbidity, HCPs, ICs, and FCs.
- A source-agnostic data collection system (CABIE).
- A portal to support (1) management of trials and participants and (2) clinical triage support (Subject Information Management System [SIMS]).
- Cloud-based storage and analytics system (KITE).
- Advanced analytics to provide risk assessment, support person with multimorbidity goal setting, and support person-centric care (CareAnalytics).

From the person with multimorbidity perspective, the measurement and sensing devices and CareApps are the only platform technologies that they interact with. For CN users, CareApps tailored to their requirements are their point of interaction with the platform. The full list of devices used by the person with multimorbidity is included in Textbox 1.

**Figure 3:**
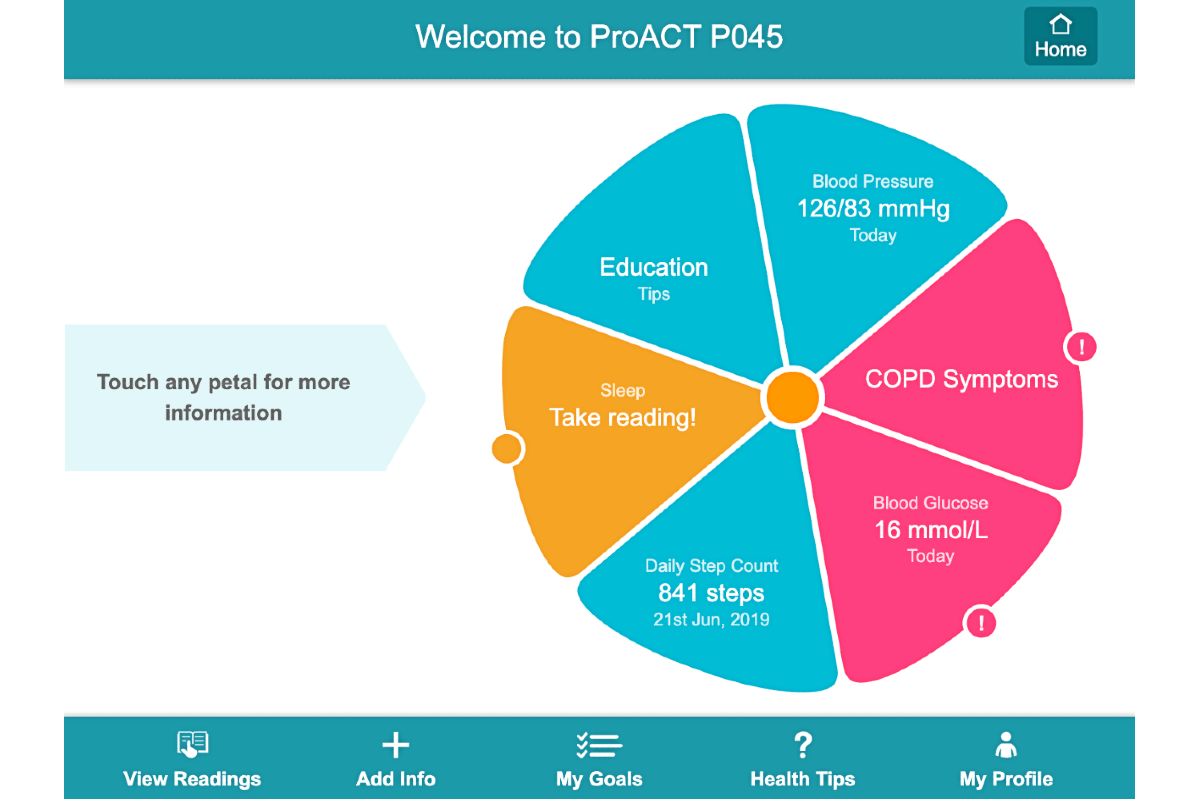
Persons with multimorbidity home screen user interface.

Within the overall intervention, the primary point of information exchange with the end user (person with multimorbidity or CN support actor) is their CareApp. Textbox 2 outlines the structure and use of each CareApp. Figure 3 provides an overview of the person with multimorbidity home screen co-designed with users. The petal-based interface presents a brief summary of health and well-being data tailored to each person with multimorbidity’s condition and self-management preferences. Using a color-coded *traffic light* system, persons with multimorbidity are alerted if their data are below or above their personal thresholds (pink), when they have not taken a reading for five days or more (orange), or when all is deemed normal for the person with multimorbidity (blue).

**Hardware or devices included in the person with multimorbidity ProACT toolkit (customizable according to the preferences and conditions of the person with multimorbidity).**

**Vital signs monitoring**

- iHealth blood glucose monitor
- Withings blood pressure monitor
- Withings weight scales
- iHealth pulse oximeter

**Well-being monitoring**

- Withings watch (physical activity and sleep)

**General**

- Tablet device (eg, iPad)
- Broadband connection (supplied where needed)
- Peripheral supplies (batteries, extension leads, etc)

**CareApp components and associated features.**

**Persons with multimorbidity**

- Home screen
  Provides a quick overview of current health and well-being status, educational tip of the day, and goal progress tailored to individual disease profiles and self-management preferences (eg, blood pressure, step count, blood glucose, and daily questions). Home button and quick links to; view readings, add info; my goals; health tips and my profile (described below).
- View Readings
  Users can choose to view their data across five key areas: symptoms, sleep, activity, daily question responses, and personal reflections on these responses.
- Add Info
  Allows for manual entry of data from personal or nondigital devices and presents daily questions around general well-being, anxiety, satisfaction with sleep, and social interactions, as well as symptom monitoring questions for those parameters not measurable by a digital device (eg, breathlessness, sputum color for chronic obstructive pulmonary disease, and edema for Heart Failure).
- My Goals
  Supports persons with multimorbidity to set personalized, flexible, and collaborative (with their care network) goals around their health and well-being (eg, exercise).
- Tips
  Tips and educational content relating to conditions and self-management (covers information related to individual conditions; managing multiple conditions; medication management; activity, social, and goal planning, etc) as well as training on how to use devices (including the iPad) and the CareApps.
- My Profile
  Supports the person with multimorbidity in having control over various aspects of their CareApp, including who they would like to share their data with and how often they would like reminders/alerts to take readings.

**Informal carer**

- The app view has a similar structure and navigation to the person with multimorbidity app. The home screen is based on a grid rather than a flower-shaped petal and presents a mixture of educational content (this includes the same content as in the person with multimorbidity app along with educational material on providing care to a person with multimorbidity, addressing topics such as self-care and time management) and person with multimorbidity health readings. The app also allows the user to send brief notifications that they have viewed the data and encourage the person with multimorbidity in their self-management practices.

**Formal carer**

- The app has the same structure and navigation as the informal carer app with similar features. This app is limited so that formal carers can only view well-being data (such as sleep and activity) and not the person with multimorbidity’s health (symptom data such as blood pressure, blood glucose, etc) readings, as this was not allowed due to regulations within the formal care organizations at trial locations.

**Health care professionals**

- The health care professional (HCP) CareApp has similar functionality to the formal carer CareApp in that HCPs can view a list of their patients and with permission, view their readings and their profile. Within this app, HCPs have access to the patient health (eg, symptom data) readings.

## Methods

### Study Design

The study was a longitudinal (12-month) PoC trial using an action research design and mixed methods approach. Action research is a period of investigation that *describes, interprets, and explains social situations while executing a change intervention aimed at improvement and involvement* [22]. The strength of this approach is the capability to generate solutions to practical problems, while garnering methods to understand the context of care, needs, and experiences of the person with multimorbidity group, drawing upon a range of research methods (eg, participant observation and in-depth interviews), to involve and build relationships with persons with multimorbidity and associated CN support actors. Within the PoC trial, this allowed for modifications to the technology based on quantitative and qualitative data collected from platform use statistics (eg, how often participants engage with the platform), platform data (ie, data coming from sensors and technologies), observational and usability testing methods to understand participant interaction with CareApps; person with multimorbidity and CN responses to interviews, questionnaires, and standardized assessments (eg, to evaluate quality of life, device proficiency, and usability).

### Participant Inclusion and Exclusion Criteria

Persons with multimorbidity and their CNs (consisting of ICs, FCs, and HCPs) were eligible for this study. For inclusion, participants with multimorbidity were aged ≥65 years and had at least two of the following conditions: diabetes, chronic obstructive pulmonary disease (COPD), chronic heart failure (CHF), or chronic heart disease or coronary artery disease including hypertension, atherosclerosis, angina, and arrhythmia; were capable of giving written informed consent; had access to broadband services (this refers to regional infrastructure); or lived in an area with sufficient coverage for mobile broadband or internet. The implemented service costs were covered as part of the trial.

CN participants were invited only on the permission of the person with multimorbidity and were required to be aged ≥18 years; to be providing care or support to a participant with multimorbidity; have access to a computer, tablet, or smartphone with an internet connection; and to be capable of giving written informed consent.

### Sample

A purposive sample of 120 persons with multimorbidity (60 persons with multimorbidity per trial site in Ireland and Belgium) were recruited to participate in the PoC trial. Although sample size is often cited as a key factor in determining the potential success of a study, this is more relevant for randomized controlled trial studies that seek to answer specific questions regarding the efficacy of interventions (does it work?) and is less relevant for studies related to care and service improvement (how does it work?) [23]. Thus, to determine the PoC sample size, we took a pragmatic approach and reviewed two important factors: (1) it is large enough to provide a reliable analysis of the ecosystem and (2) small enough to be financially feasible. An analysis of the literature suggests that the overall sample size in a PoC, telemedicine and health focused information communications technology trial is low. A review of 1030 studies on telemedicine-based technological interventions for chronic disease management, looking at CHF (436 studies), stroke (422 studies), and COPD (172 studies) between 2005 and 2013 (including 35 systematic reviews and one review of the reviews), suggested that methodologically robust sample sizes for each condition were 17 participants (COPD), 21 participants (stroke), and 19 participants (CHF) [24]. The selected studies were conducted primarily in the United States and Europe.

### Ethical Approval and Consent

Ethical approval was granted from participating health service organizations where recruitment took place and from academic partners. Informed consent was obtained on an individual basis in accordance with legal and ethical guidelines at each trial site region, following careful explanation of the study and provision of participant information and informed consent forms for the person with multimorbidity and participating members of their CN. All participants had the right to withdraw from the study at any time without any questions. Following a review of recruitment procedures by ethical committees in Ireland and Belgium, it was agreed that researchers should only contact a person’s HCP if they had provided this consent.

### Recruitment Procedures

In both Ireland and Belgium, participants were selected by several different methods, depending on which recruitment source they were accessed through, as outlined below:

- HCP and FC services (eg, in Ireland, the Health Service Executive and Home Instead Senior Care, and in Belgium, the hospitals UZGent and OLV Aalst, and the home care organizations Solidariteit voor het Gezin and Rivierenland): participants were selected from the service clinic records or via professional familiarity by HCPs employed directly in the services; HCPs within the services selected any potential participants who met the study inclusion criteria. Research team members did not view health service records to identify participants.
- ProACT *requirements gathering* panel: this research panel consisted of individuals linked to the first phase of the ProACT project, which focused on the design and development of the platform. Phase 1 received ethical approval, and participants consented to be recontacted regarding participation in the PoC trial.
- General practices: participants were selected by general practitioners (GPs) following the same procedures outlined for health professional services. Study information was also left in participating GP waiting rooms. Self-selecting participants who viewed this information could then directly contact the research team. Researchers assessed potential participants to determine whether they met the inclusion criteria (eg, whether they have been diagnosed with the ProACT conditions). If they were unsure, they were asked to check with their GP.
- Relevant older persons and chronic disease networks (eg, diabetes and COPD support groups): participants were self-selected. These organizations disseminated study information to their members, who could then directly contact the research team to participate. The same assessment procedures outlined for general practices were applied.
- Additional recruitment sources in Ireland included social media, radio, and local newspaper advertising; referrals directly from pharmacists; and participants who also referred another person with multimorbidity. Researchers contacted individuals who expressed interest in participating to ensure that they met the inclusion criteria.
- Additional recruitment sources in Belgium included several recruitment agencies (IVOX, Tendens, imec Living Lab, and Zorglab Aalst) via their respective panels, a pharmacy organization, a newspaper advertisement, and participants who also referred another Person with multimorbidity.
- In relation to the additional recruitment channels in Ireland and Belgium, researchers assessed potential participants to determine whether they met the inclusion criteria (eg, whether they had been diagnosed with the ProACT conditions). If they were unsure, they were asked to check with their GP.

### Technology Deployment and Trial Setup

Invited person with multimorbidity participants had at least 7 days to review the participant information leaflet and have queries answered before technology deployment, which occurred over 2 visits to the person with multimorbidity’s home. All researchers ensured that ProACT technology was deployed correctly and in a consistent manner across trial sites, following a strict deployment plan.

During the first visit, members of the research team obtained written consent from the participants. Each participant received devices depending on their condition profile. Participants also had the option to use any existing device (that they currently use at home) to measure an included parameter (eg, blood glucose monitor) by manually entering readings from the device into the person with multimorbidity CareApp. ProACT sensor devices were connected by Wi-Fi or Bluetooth, and a broadband internet connection was provided for the duration of the trial for any participants who did not have existing broadband in their homes. Participants were trained on how to use their ProACT devices during their initial visit. This included a brief introduction on how to use the ProACT CareApp and associated third party apps (eg, using the Withings HealthMate [25] app to take a blood pressure reading), as it was important that the person with multimorbidity was not overloaded with information on all ProACT technology features during the first visit. Participants were also provided with a paper-based manual, containing detailed instructions for using each device, along with common troubleshooting instructions.

Approximately 1 week after the first visit, the researchers conducted a second deployment visit. Detailed training on the CareApp took place with additional web-based training materials and videos made available through the ProACT CareApp. A study helpdesk, staffed by respective research team members in Ireland and Belgium, was available (from 9:30 AM- 4:30 PM, Monday to Friday) to assist participants with queries and technical difficulties. In both Ireland and Belgium, a dedicated clinical triage service for monitoring vital signs was also available (9 AM-5 PM, Monday to Friday). Triage personnel (clinical nursing staff) had access to data from all persons with multimorbidities participating in the trial via the SIMS. A protocol for dealing with potential adverse events was developed using triage personnel. This included defining thresholds for abnormal vital sign values for each parameter being monitored. For example, thresholds for high and low blood glucose values were set in the SIMS for participants with diabetes. At the outset of the trial, global threshold values were set for all participants. However, over the course of the trial, such thresholds were often adjusted for individuals based on their normal values. If a participant’s vital sign reading is outside the normal threshold, an alert is triggered on the SIMS triage interface, and as noted above, the participant will see a pink petal on their CareApp dashboard (Figure 3). In such instances, the triage nurse calls the participant to discuss the reading and determine whether an escalation is required. In both trial regions, clinical triage was not provided for nonvital sign data (eg, sleep or activity). Participants were reminded that this was a research study and that the triage service would not be considered as a replacement for normal care. In the event that a person with multimorbidity felt ill, they were recommended to seek medical advice or care as they normally would. Persons with multimorbidity were also reminded of this at regular intervals through a pop-up message on their CareApp, as requested by the ethics committees. Following completion of the second deployment visit, the participants began their trial period.

Invited members of the person with multimorbidity’s CN were provided with access to their relevant CareApp that they could use on their own devices (smartphones, tablets, or computers). These customized CareApps allowed those in the CN to view relevant data from the participant with multimorbidity and educational materials related to condition management, well-being, and technology use. Participants with multimorbidity chose what data to share with each CN participant. The data viewed by HCP participants via their CareApp were not used to make clinical decisions. This was clearly outlined in the participant consent forms and information leaflets for all trial participants.

### Trial Implementation, Outcome Measures, and Data Collection

#### Overview

**Figure 4:**
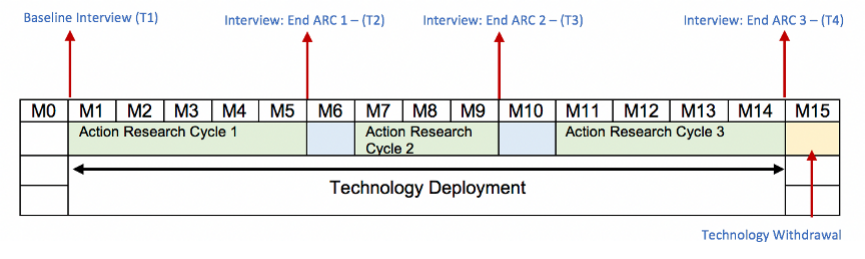
Study timeline across action research cycles for persons with multimorbidity.

The person with multimorbidity CareApp and toolkit were deployed to the person with multimorbidity in their homes for up to 12 months (participants used the app for a minimum of 9 months to cover the three action research cycles), across a 15-month period. Recruitment was staggered across action research cycle 1, as outlined in Figure 4. Introducing participants at various stages in the first action research cycle did not impact the final analysis, as elements of the system were redesigned or developed at 2 separate points, as part of the action research methodology. Invited CN participants also received access to their respective CareApp following nomination from the person with multimorbidity. Outcomes from the trial were assessed using a mix of ProACT platform data (engagement with app and data from sensors), CareApp questionnaires (self-report data on health and well-being), standardized assessments (Table 1), usability testing, and semistructured interviews. Further details of the process for the person with multimorbidity and CN members are shown in Table 1.

**Table 1:** Persons with multimorbidity key assessment domains and measures.^a^

Domain	Scale or measure	Description of measure	Assessment time point
Demographics	Self-report questionnaire^b^	7 self-report items collecting information on gender; date of birth; marital status; educational level; living alone or with others; employment status; primary occupation	T1
Medication list	Self-report list	Interviewer recorded a list of the names, dosage, and frequency of each participant’s medications. These data were used to initially populate the triage system for nurses, who then managed the ongoing collection and updated medication information	T1
Comorbidity index/disease burden	Multimorbidity assessment by self-report [26]^b^	22-item list of common conditions or comorbidities: yes or no to indicate presence of conditions; then 5-point Likert scale to assess the extent to which each condition limits daily activities	T1 and T4
Technology use and proficiency	Mobile device proficiency questionnaire [27]^b^	16-item scale to assess older adults’ proficiency with mobile technological devices. Participant-rated ability to carry out different operations (internet, calendar, etc) on a 5-point Likert scale	T1 and T4
Cognitive function	Montreal cognitive assessment [28]	30-item scale measuring cognitive function in several domains; total score gives measure of global cognition; cognitive screening test	T1 and T4
Health related quality of life/health outcome measure	The 5-level EuroQol-5D version [29]	5-item self-report Likert scale: rate level of problems in five dimensions: mobility, self-care, usual activities, pain or discomfort, and anxiety or depression 1-item visual analogue scale: own judgment of health status between 1 and 100 (from “best health you can imagine” to “worst health you can imagine”)	T1, T2, T3, and T4
Quality of life	Control, Autonomy, Self-Realization and Pleasure-19 [30]	19-item scale measuring quality of life across four dimensions: control, autonomy, pleasure, and self-realization. Developed for an older adult population.	T1, T2, T3, and T4
Illness perceptions	Multimorbidity Illness Perceptions Scale [31]^b^	22-item scale measuring illness perceptions related to multimorbidity in five dimensions: emotional representations, treatment burden, prioritizing conditions, causal links, and activity limitation	T1, T2, T3, and T4
Self-efficacy	General self-efficacy scale [32]	10-item self-report Likert scale: assesses perceived self-efficacy and ability to cope with daily hassles and stressful life events	T1, T2, T3, and T4
Locus of control	Multidimensional health locus of control scale [33]	18-item scale assessing beliefs about control individuals have over their own health in three main dimensions: internal control, chance, and power	T1, T2, T3, and T4
Social connectedness	Lubben social network scale [34]	18-item version to measure social connection in three domains: family, friends, and neighbors	T1 and T4 (18 item); T2 and T3 (6 item)
Depression and anxiety	Hospital Anxiety and Depression Scale [35]	14-item scale to measure depression and anxiety—developed as a screening tool for clinical levels of depression and anxiety	T1 and T4
Sleep quality	Pittsburgh Sleep Quality Scale [36]	9-item scale to assess subjective sleep quality: can provide an overall score and domain specific scores	T1 and T4
Fatigue	Functional Assessment of Chronic Illness Therapy Fatigue Scale [37]^b^	13-item scale measuring feelings of fatigue, weakness, or energy and impact on daily activities	T1 and T4
Physical activity	Rapid Assessment of Physical Activity [38]	10-item scale to measure engagement in physical activities	T1 and T4
Usability	System Usability Scale [39]	10-item scale (Likert scale item) to provide subjective assessment of the usability of a technology system	T2, T3, and T4
User burden (technology)	User Burden Scale [40]^b^	18-item^c^ self-report scale used to evaluate user burden when engaging with technology. Likert scale	T2, T3, and T4

^a^Measures administered at each assessment time point are a subset of those listed in this table; an indication of the time point for each assessment is indicated in the table below.

^b^These measures were included as part of a paper-based questionnaire sent to participants in advance of the relevant interview.

^c^The original questionnaire has 20 items, but two questions in relation to financial burden were not used due to lack of relevance.

#### Person With Multimorbidity

Participants with multimorbidity participants were asked to use their CareApp to record information and measure key parameters related to their health and well-being on a regular basis (at their convenience), using sensors/devices and by answering self-report questions presented via the CareApp. They could also use their CareApp to view their recorded data and view educational materials and training videos related to condition management, well-being, and technology use. Adherence to physiological monitoring and use of the ProACT CareApp was monitored via system use statistics and data collected by the ProACT platform.

The persons with multimorbidity’s questionnaire or assessment and qualitative semistructured interview data were collected across four time points: baseline (T1 during second deployment visit), at the end of each 3-month action research cycle (T2: month 3; T3: month 7), and in a final posttrial interview (T4: month 12). Figure 4 presents the study timeline for the persons with multimorbidity.

A paper questionnaire containing scales and measures suitable for self-completion was posted to each participant before each interview. This allowed the participant to complete these measures at a time that was convenient to them to reduce participant burden. Interviews were conducted at the participants’ homes. The researchers reviewed the questionnaires briefly during interviews and assisted the participants in completing any questions where necessary. Table 2 presents the key assessment domains and measures issued to Persons with multimorbidity across the trial. Semistructured qualitative interviews were also conducted. Themes that were addressed in the interviews included understanding expectations of how ProACT might change health and well-being; persons with multimorbidity’s use of ProACT; understanding how ProACT has changed self-management routines or strategies; the impact of ProACT on the role of the CN; frequency of health care use and cost of care; accessibility and usability of ProACT; user satisfaction and effectiveness of ProACT; and technology adoption and perceived future use of ProACT.

Following action research cycle 3, the trial concluded with a 1-month period of phased withdrawal of the technology. The timeline for the withdrawal of the technology was clearly explained to the participants throughout the study to manage participant expectations.

**Table 2:** Care network participant key assessment domains and measures.

Domain	Measure	Time point	Who
Demographics	Self-report items^a,b^ ICsc: age, gender, education, relationship with persons with multimorbidity, employment status, primary occupation, hours and type of care, and self-rated health FCsd and HCPse: age, gender, duration of care provided to persons with multimorbidity, and type of care provided to persons with multimorbidity	T1 only	IC^c^, FC^d^, and HCP^e^
Technology use and proficiency	The Mobile Device Proficiency Questionnaire^a^ [27]^b^	T1 and T4	IC and FC
Usability	System Usability Scale [39]	T4 (with a subset only)	IC and FC
User burden (technology)	User Burden Scale^a^ [40]^b^	T4 (with a subset only)	IC and FC
Self-efficacy	General Self-Efficacy Scale^a^ [32]^b^	T1 and T4	IC and FC
Stress	Perceived Stress Scale [41]: 14-item scale of the degree to which situations in an individual’s life are appraised as stressful	T1 and T4	IC and FC
Caregiver stress or psychological impact of caregiving	Caregiver Self-Assessment Questionnaire [42]: 18-item scale to measure the psychological impact (including stress) of caregivers	T1 and T4	IC
Caregiver burden	Zarit Burden Interview [43]: 22-item scale to measure the level of burden experienced by caregivers of patients	T1 and T4	IC

^a^Measures included as part of a paper-based questionnaire sent to participants in advance of the relevant interview.

^b^These measures were included as part of a paper-based questionnaire sent to participants in advance of the relevant interview.

^c^IC: informal carer.

^d^FC: formal carer.

^e^HCP: health care professional.

To assess whether the ProACT CareApps were usable and accessible, we conducted user evaluations with a small subset of users over repeated time points (in line with the action research cycles) during the trial. Participants were asked to conduct a number of tasks and give their opinions and feedback on the app using a *think-aloud* protocol [44]. This involves encouraging participants to verbalize what they are thinking as they use the app to expose potential usability and accessibility issues. Users were video-recorded during the evaluations. The resulting videos were transcribed, annotated, and analyzed by researchers to explore participant interactions with the technology and identify any barriers or difficulties that they encountered. The results of these evaluations were used to update the CareApp interfaces during the trial to enhance the usability and accessibility of the app.

#### Care Network

##### Overview

Consenting CN participants came to the trial during the person with multimorbidity’s ARC 2 based on referrals from persons with multimorbidity during ARC 1. All users in the CN were provided with relevant data for the participant with multimorbidity participant and relevant training or educational content via their customized ProACT CareApp. These data could be viewed at a time and frequency that was convenient for them. The purpose was to evaluate the experiences of people within the CN using the ProACT platform and to understand whether they would find this type of system and data useful to them in their role, supporting the person with multimorbidity with his/her self-management, care, and treatment plans. Members of the research team collected feedback and evaluation data from people in the CN, as described in the following sections:

##### Informal Carers

A member of the research team conducted interviews with ICs, either by phone or at a location convenient to the participant at T1 (ie, when the CN participant consented to take part) and T4 (at the end of the trial). While a person with multimorbidity could have more than one IC in their CN who had access to the CareApp, only one, the primary IC, was asked to complete the assessments or interviews. During this interview, the researcher administered scales and questionnaires to collect information on health, psychosocial, psychological, and demographic characteristics (Table 2). A semistructured, qualitative interview was also conducted, covering areas including expectations of the use of ProACT, usability of the CareApp, whether ProACT has benefitted them in their role, and how they felt it benefited the person with multimorbidity. ICs were also asked to complete a short questionnaire to provide feedback on the technology at the end of the trial (T4).

##### FCs and HCPs

Participants were asked to complete a short questionnaire at T1 (ie, when the CN participant consented to take part) and T4 (at the end of the trial). These questionnaires collected information on the usability and acceptability of the technology, along with experiences of using the ProACT platform (Table 2). FC and HCP participants also participated in qualitative interviews or focus groups at baseline (T1) and posttrial (T4). Themes addressed were whether ProACT helped in their role, how they felt it benefitted the person with multimorbidity, what would they change about the system, and the usability of the CareApps.

### Data Analysis

As a PoC trial, a key outcome is to understand whether a larger trial that makes a definitive assessment of benefit is warranted. Pilot and PoC studies are more about learning than confirming or formally assessing evidence of the impact or benefit associated with an intervention. Therefore, analyses should focus on providing descriptive evidence and indications of the range of possible responses rather than on formal hypothesis testing [45]. Analyses were therefore mainly descriptive and aimed at understanding user experiences in relation to the use of the ProACT platform. Qualitative methods encouraged participants to speak about their experiences of living with and managing multimorbidity and their experience of using ProACT technologies. Quantitative data analysis ensured comparability and consistency of questions across participants and time points.

Qualitative data were analyzed using thematic analysis (TA) to identify and understand emerging themes. An inductive approach was adopted to identify themes at a latent level. An inductive TA is data-driven, as opposed to analyst-driven TA [46]. This approach helps generate novel insights from interview data that may have differed greatly from pre-existing research in the area pertaining to the research questions. This is essential to the action research design of the trial to analyze differences in responses across time points. Furthermore, identifying themes at a latent or interpretative level goes beyond the semantic meaning of the presented data, encouraging interpretative analysis by the researchers. Across the PoC trial locations, a protocol (including in-person and web-based training) was put in place to ensure that the TA followed a strict analytical process, with researchers ensuring transparency and consensus across each step. Individual researchers coded the transcripts according to an established analysis protocol. Pairs of researchers collapsed and categorized codes into themes. Discussions and recoding workshops were conducted to ensure agreement on theme and subtheme names were reached among the wider trial site teams. In Ireland, NVivo for Mac (version 11; QSR International) [47] was used to conduct the coding part of the analysis, while in Belgium, MAXQDA Analytics Pro (VERBI GmbH; [48]) was used. Using different software did not impact the analysis, as the same methodological approach was used at both sites.

Quantitative questionnaire data were analyzed at both trial sites using SPSS statistical software (version 25; IBM SPSS Statistics [49]). The primary analysis was to evaluate changes in scores between the assessment points. Descriptive statistics were used to summarize participant demographic data and general outcomes from the questionnaire data. Sensitivity analysis was performed to treat missing data. Missing data were imputed based on the methods suggested for each questionnaire. In case a standardized method was not reported in the literature, mean substitution, using similar imputations for all questionnaires, was used for all time points, if less than 20% of the data were missing. Initial analyses were conducted to assess the distribution of all variables and check for relevant assumptions, including normality. Given the small sample size at each trial site, the majority of variables violated normality. Therefore, to maintain the intrinsic value of the quantitative data in this circumstance, no transformations were performed, and for further inferential analysis, nonparametric (Friedman and Wilcoxon signed-rank) tests were implemented.

The SIMS component of the ProACT platform supported the analysis of additional data (including sensor data from the devices and engagement with the devices and ProACT CareApps). Metrics of interest for analysis included symptom (eg, blood pressure, blood glucose, SpO_2_, and weight) trends or patterns over time; the ratio of alerts to symptom readings over time; trends or patterns in activity and sleep data over time and engagement with various parts or features of ProACT and the CareApp; and responses to self-report questions on health and well-being.

## Results

This was a 44-month funded study (2016-2019). The implementation phase was completed in June 2019. In total, 120 persons with multimorbidity (60/120, 50% in Ireland and 60/120, 50% in Belgium) and 73 CN participants (43/73, 59% in Ireland and 30/73, 41% in Belgium) were recruited. The trial outcomes are at various stages in the process of publication from 2021. We believe that the ProACT platform can potentially improve how older adults with multimorbidity self-manage their health and well-being from home, supported by their CN.

## Discussion

### Summary

Across the EU, there is a growing drive to meet the complex care needs of older people with multimorbidity. eHealth or digital health options are now recognized as potential support [22]. However, EU health care systems are not yet equipped to address the comprehensive care needs of people with multimorbidity [50]. The use of innovative person-centered digital health technologies are increasingly viewed as a means to address the challenge of multimorbid care (eg, tools to support patients’ self-management and multidisciplinary collaboration between professionals [51] may play a key role in advancing the integration of health and social care needs). Despite this, research into the design and development of digital health systems, focusing on multimorbidity management, particularly for older adults, is in its infancy.

It is important to re-emphasize that the focus of this research is on multimorbidity (multiple co-occurring chronic conditions, but with a focus on multiple conditions) as opposed to comorbidity (multiple co-occurring chronic conditions, but with a focus on a singular condition) [52], which seeks to advance a multi-country understanding of the challenges for defining, designing, implementing, and evaluating a digital intervention, focused primarily on multimorbidity management across diverse populations. To our knowledge, ProACT is also the first digital intervention to systematically incorporate (and evaluate) behavioral change and human-computer interaction methods to advance persons with multimorbidity’s self-management practices in relation to multimorbidity.

With the mixed methods, action research PoC study of the ProACT platform, we are further addressing the need for increased longitudinal and applied research in the areas of digital health, integrated care, and multimorbidity management. The two primary aims of ProACT are as follows:

- To explore the potential benefits of technological support (ie, the ProACT platform) that aim to improve integrated care and self-management practices for older persons with multimorbidity.
- To obtain feedback from all relevant participant groups on their experiences using the ProACT platform and on the potential for the ProACT platform to improve integration of care and support disease management for older persons with multimorbidity.

Outcomes from trials [53] are positive in terms of user engagement with ProACT and a shift in behavior to adopt this digital intervention. These outcomes will help advance both the state of the art on how to design and conduct research with older persons with multimorbidity and their CN and deliver a new digital health solution to address the challenge of multimorbidity management and care.

### Conclusions

Although substantial research has been conducted on the implementation and use of digital health technologies to address single-disease management, a clear gap exists in understanding the requirements for managing multimorbidity from the perspective of older persons with multimorbidity and their CN and how supported self-management happens in practice. The findings from the ProACT PoC trials will contribute significantly to the research in this field. With 120 older persons with multimorbidity and 73 CN participants, the trials have provided a novel multi-stakeholder, multi-country perspective on multimorbidity self-management and integrated care. With a primary focus on qualitative outcomes, the PoC trials have provided detailed insight into the person with multimorbidity’s self-management journey facilitated by a digital health platform, longitudinally over 12 months. Outcomes will evaluate the impact of ProACT at a PoC level to determine whether a larger trial, which makes a definitive assessment of benefit, is warranted.

## Appendix

Multimedia Appendix 1 ProACT Evaluation Summary Report European Commission.

## Abbreviations

CHF: chronic heart failure
CN: care network
COPD: chronic obstructive pulmonary disease
EU: European Union
FC: formal carer
GP: general practitioner
HCP: health care professional
IC: informal carer
PoC: proof-of-concept
SIMS: subject information management system
TA: thematic analysis
