# Supplementary material for: A Digital Health Platform for Integrated and Proactive Patient-Centered Multimorbidity Self-management and Care (ProACT): Protocol for an Action Research Proof-of-Concept Trial
Source: JMIR Res Protoc. 2021 Dec 15;10(12):e22125. doi: 10.2196/22125 (PMC8717136; doi:10.2196/22125)
Supplement: Multimedia Appendix 1 [file resprot_v10i12e22125_app1.pdf]

Proposal Evaluation Form

EUROPEAN COMMISSION

Horizon 2020 - Research and Innovation Framework Programme

Evaluation Summary Report - Research and innovation actions/Innovation actions

**Call:**	H2020-PHC-2015-single-stage
**Funding scheme:**	Research and Innovation action
**Proposal number:**	689996
**Proposal acronym:**	ProACT
**Duration (months):**	42
**Proposal title:**	Integrated Technology Ecosystem for ProACTive Patient Centred Care
**Activity:**	PHC-25-2015 Integrated care RIA

N.	Proposer name	Country	Total Cost	%	Grant Requested	%
1	THE PROVOST, FELLOWS, FOUNDATION SCHOLARS & THE OTHER MEMBERS OF BOARD OF THE COLLEGE OF THE HOLY & UNDIVIDED TRINITY OF QUEEN ELIZABETH NEAR DUBLIN	IE	709,800	14.55%	709,800	14.55%
2	IBM IRELAND LIMITED	IE	682,506	14.00%	682,506	14.00%
3	ASSOCIAZIONE ITALIANA PER L ASSISTENZA AGLI SPASTICI PROVINCIA DI BOLOGNA	IT	447,500	9.18%	447,500	9.18%
4	DUNDALK INSTITUTE OF TECHNOLOGY	IE	789,026	16.18%	789,026	16.18%
5	IMINDS VZW	BE	700,375	14.36%	700,375	14.36%
6	UNIVERSITY COLLEGE CORK, NATIONAL UNIVERSITY OF IRELAND, CORK	IE	260,520	5.34%	260,520	5.34%
7	TREELOGIC TELEMATICA Y LOGICA RACIONAL PARA LA EMPRESA EUROPEA SL	ES	311,250	6.38%	311,250	6.38%
8	PHILIPS ELECTRONICS UK LIMITED	UK	663,853	13.61%	663,853	13.61%
9	Home Instead Franchising Ltd.	IE	109,375	2.24%	109,375	2.24%
10	VEREIN ZUR FOERDERUNG ASSISTIERENDER TECHNOLOGIE IN EUROPA	AT	69,375	1.42%	69,375	1.42%
11	EUROPEAN ASSOCIATION OF SERVICE PROVIDERS FOR PERSONS WITH DISABILITIES AISBL*ASSOCIATION EUROPEENNE DES PROMOTEURS DE SERVICES POUR DES PERSONNES AVEC UN HANDICAP	BE	70,050	1.44%	70,050	1.44%
12	Asp Città di Bologna	IT	63,125	1.29%	63,125	1.29%
	Total:		4,876,755		4,876,755	

Abstract:
ProACT targets Europe’s 50 million multimorbid patients to proactively self-manage and offset the EU’s annual €700billion cost of chronic disease management. ProACT aims at providing and evaluating an open application programming interface to integrate a variety of new and existing technologies to advance ‘home based’ integrated care (IC). Cloud based data analytics will determine correlations between technology use and the influence of support actors to impact on the health and quality of life of patients. Research will examine 4 models of care/support, central to implementing effective, continued and coordinated patient-centric care/self-management. Development of a novel data aggregation and cloud platform system will enable data analysis for improvement of IC, effective measurement of results and comparison of efficiency and costs, so that the relationship between patients and their personalized care network is optimized. Proof of concept trials (120 patients in total, with associated care/support actors) will be carried out within Health Services (Ireland and Belgium) with associated living lab facilities to ensure patient co-design technology approaches. Clinical status information, therapies and activity tools will be deployed for the conditions of: chronic heart failure (CHF), diabetes and chronic obstructive pulmonary disease (COPD). Tools to support mild cognitive impairment and detect early onset dementia are included. Commercial potential will be validated during the project supported by a European feasibility study to assess the cultural and political determinants for adoption and scalability of the ecosystem. ProACT engages a multidisciplinary EU consortium of 3 public and 9 private organizations (including 2 of the world's leading ICT companies, the largest home care provider and 2 EU service provider and technology networks) to develop and validate the ecosystem. Individually partners could develop the components. Together we can develop the system.

Evaluation Summary Report

Evaluation Result

Total score: 14.50 (Threshold: 12)

Form information

SCORING

Scores must be in the range 0-5.

Interpretation of the score:

0 *The* ***proposal fails to address the criterion*** *or cannot be assessed due to missing or incomplete information.*

1 Poor. *The criterion is inadequately addressed, or there are serious inherent weaknesses.*

2 Fair. *The proposal broadly addresses the criterion, but there are significant weaknesses.*

3 Good. *The proposal addresses the criterion well, but a number of shortcomings are present.*

4 Very good. *The proposal addresses the criterion very well, but a small number of shortcomings are present.*

5 Excellent. *The proposal successfully addresses all relevant aspects of the criterion.Any shortcomings are minor.*

Criterion 1 - Excellence

Score: **5.00** (Threshold: 4/5.00 , Weight: 100.00%)

Note: The following aspects will be taken into account, to the extent that the proposed work corresponds to the topic description in the work programme. If a proposal is partly out of scope, this must be reflected in the scoring, and explained in the comments.
Clarity and pertinence of the objectives
Credibility of the proposed approach
Soundness of the concept, including trans-disciplinary considerations, where relevant
Extent that proposed work is ambitious, has innovation potential, and is beyond the state of the art (e.g. ground-breaking objectives, novel concepts and approaches)

This is a highly ambitious proposal with very clear and aligned objectives targeting all aspects of this very complicated space. The target group is well described and appropriate.

The proposal is very credible and addresses all the relevant issues using an effective iterative approach. A comprehensive and clear understanding of the relevant barriers is very evident. Well defined trials with clear outcome assessment measures demonstrate a sound approach to proof of concept.

The concept is sound, effectively addressing both the system architecture of an integrated health and social care system and the interface of the equipment in the home of the user.

A solid understanding of the relevant state of the art is well evidenced, with the proposal building on projects such as CABIE. The proposal clearly demonstrates the application of state of the art techniques in all relevant aspects, from ICT to process design to change management with credible intentions to progress beyond current levels. The result is a novel and thorough response to the call and the underlying societal need.

Criterion 2 - Impact

Score: **5.00** (Threshold: 4/5.00 , Weight: 100.00%)

Note: The following aspects will be taken into account, to the extent to which the outputs of the project should contribute at the European and/or International level:
The expected impacts listed in the work programme under the relevant topic
Enhancing innovation capacity and integration of new knowledge
Strengthening the competitiveness and growth of companies by developing innovations meeting the needs of European and global markets, and where relevant, by delivering such innovations to the markets
Any other environmental and socially important impacts (not already covered above)
Effectiveness of the proposed measures to exploit and disseminate the project results (including management of IPR), to communicate the project, and to manage research data where relevant

The proposal provides a coherent and credible analysis of all impacts in relation to the call, with realistic explanations of how they will be achieved. Relevant and appropriate, mostly qualitative, metrics are presented.

A clear intention to enhance innovation capacity is evident, for example, through the use of an open innovation model to encourage contributions from third party developers. Transferability is also explicitly addressed within one of the pilots.

Opportunities to strengthen competitiveness and support growth are well presented and convincing. A comprehensive market analysis, very clear and credible proposed business measures and the active engagement of major industry stakeholders gives confidence in the exploitation potential.

The dissemination and communication plans are comprehensive and very appropriate, with good key performance indicators. Exploitation plans are very sound. IPR issues are suitably addressed.

Criterion 3 - Quality and efficiency of the implementation

Score: **4.50** (Threshold: 3/5.00 , Weight: 100.00%)

Note: The following aspects will be taken into account:
Coherence and effectiveness of the work plan, including appropriateness of the allocation of tasks and resources
Complementarity of the participants within the consortium (when relevant)
Appropriateness of the management structures and procedures, including risk and innovation management

This is a well presented work plan with coherent work packages, detailed tasks that are appropriately allocated, clearly structured to deliver the objectives of the proposal. Resource allocation is sound and appropriate.

The consortium is very well balanced and complementary with relevant stakeholders all present. The proposal offers clear evidence of the expertise necessary to undertake the tasks.

The management structure and procedures are well described and relevant for this complex proposal. Risks are clearly identified and appropriate, although the likelihood of the risk of users not accepting solutions is underestimated and the consequent mitigation measures are not sufficiently detailed.

Operational Capacity

Status: **Operational Capacity: Yes**

Not provided

Proposal content corresponds, wholly or in part, to the topic description against which it is submitted, in the relevant work programme part

Status: **Yes**

Not provided

Overall comments

Not provided
